# Detecting Epistatic Interactions in Metagenome-Wide Association Studies by metaBOOST

**DOI:** 10.1155/2014/398147

**Published:** 2014-07-24

**Authors:** Mengmeng Wu, Rui Jiang

**Affiliations:** MOE Key Laboratory of Bioinformatics, Bioinformatics Division and Center for Synthetic & Systems Biology, TNLIST, Department of Automation, Tsinghua University, Beijing 100084, China

## Abstract

*Material and Methods.* We recall the definition of epistasis and extend it for metagenomic biomarkers and then we describe the overview of our method metaBOOST and provide detailed information about each step of metaBOOST. *Results.* We describe the data sources for both simulation studies and real metagenomic datasets. Then, we describe the procedure of simulation studies and provide results for it. After that, we conduct real datasets studies and report the results. *Conclusions and Discussion.* Finally, we conclude our method and discuss some possible improvements for the future.

## 1. Introduction

The importance of microbial communities to human health has been attracting more and more attention, with examples including the reveal of associations between intestinal microbiome and Crohn's disease [1], the study of the relationships between gut microbiome and Type II diabetes [2], and many others [3–5]. In traditional studies of microbial communities, individual microorganisms should be cultured and sequenced separately. Although this method has successfully yielded thousands of complete bacterial genomes as recorded in GenBank [6], such limitations as the requirement of culturing individual microbes have greatly restricted the scope of applications of such a traditional approach. With recent advances of high throughput sequencing techniques, the acquirement of a microbial community has become a routine, resulting in the boom of metagenomics [6–9].

Particularly, the application of metagenomics to the study of human complex diseases has yielded the so-called metagenome-wide association (MGWA) studies [2]. For example, Qin et al. developed a two-stage MGWA study to explore relationships between gut microbiome and type II diabetes [2, 10]. Such studies have also revealed associations between intestinal microbiome and Crohn's disease [1, 3, 11] and obesity [12]. Typically, the goal of a MGWA study is to identify metagenomic markers in both genetic and functional levels, and the typical procedure of a MGWA study include three main steps. First, microbial communities for both case and control individuals are sequenced, and resulting reads are assembled to obtain a microbial gene scaffold. Second, sequence reads are mapped back to the gene scaffold, and abundance levels of microbial genes are estimated. Third, microbial genes are further mapped to known microbial organisms or a gene category, and abundance levels of organisms or gene groups are obtained. Forth, statistical or machine learning methods are applied to analyze abundance levels for both the case and the control data, and candidate microbial markers (either organisms or genes) are identified. Finally, in the marker validation phase, additional samples are sequenced to confirm the discovered markers.

Although a MGWA study has supplied us with a powerful way to search metagenomic markers associated with a disease under investigation, this approach may encounter similar problems as the traditional genome-wide association (GWA) studies. For example, it is believed that a complex disease is typically caused by multiple genetic factors, their interactions, or their interactive effects with environmental factors [13]. Particularly, interactions between genetic factors, typically referred to as epistatic interactions or epistasis, have been believed as a common pathogenic mechanism for complex diseases. However, the detection of epistatic interactions for a GWA study is extremely difficult, due to the fact of that the underlying mechanism of epistatic interaction is largely unknown and the number of possible combinations of genetic factors is typically huge. Facing this challenge, a number of computational methods have been developed. For example, Nelson et al. proposed a combinatorial partitioning method to exhaustively search for a combinatorial genotype that had the most significant contributions to a continuous phenotype [14]. Ritchie et al. proposed a multifactor-dimensionality reduction (MDR) method in which exhaustive search was performed to detect combinations of loci with the highest classification capability [15]. Jiang et al. used a machine learning method called random forest to find combinations of genotypes that contribute most to the correct classification of case against control [16]. Zhang and Liu proposed a Bayesian partition approach called BEAM to find groups of genotypes with large posterior probability [17]. Tang et al. proposed the concept of epistatic module and designed a Gibbs sampling approach to detect such modules [18]. Strategies based on high performance computing were also designed and extended to be used with graphics processing units (GPU), yielding such highly efficient method as BOOST and GBOOST [19, 20].

With these understandings, we proposed in this paper the first study of epistatic interactions in MGWA studies. More specifically, we designed a method called metaBOOST to detect such epistatic interactions for metagenome-wide case-control data. Our method consists of three main steps: (1) inference of metagenomic abundance level, (2) detection of possible epistasis using statistical methods, and (3) validation and visualization of epistatic interactions patterns. We validated our method by using both simulated experiments and real datasets studies. Results not only demonstrate the effectiveness of our approach but also provide biological insights for the pathogenic mechanisms of microbial communities to human complex diseases.

## 2. Materials and Methods

### 2.1. Overview of metaBOOST

Studies in medical genetics have shown that epistasis, or epistatic interactions between two or more genes, widely exists in such human complex disease as diabetes [21], asthma [22], and many others [23]. Recent advances in statistical genetics have also resulted in the prosperity in computational methods for detecting epistatic interactions in GWA studies, with examples which include such statistical methods as BEAM [17] and epiMODE [18], such machine learning models as epiForest [16], and such high performance computing approaches as BOOST [19]. Nevertheless, the definition of epistasis is controversial. For example, Bateson first introduced in 1909 the concept of epistasis, referring to a masking effect that one locus prevents another locus from manifesting its effect [13]. Fisher further defined in 1918 the epistatic interaction of multiple alleles at different loci as the deviation from additivity when considering contributions of these alleles to a quantitative trait [13]. With this definition, an epistatic interaction can be characterized by a logistic regression model, as

(1) $$\begin{array}{c} \log\frac{P\left(Y=1\right)}{P\left(Y=0\right)}=\mu +\alpha X_{1}+\beta X_{2}+\gamma X_{1}X_{2}, \end{array}$$

where the response item at the left hand represents the log odds of the disease risk, *X*_1_, *X*_2_ at the right hand represent the independent effect caused by two different loci, and the multiplicative item (*X*_1_ *X*_2_) represents the epistatic interaction. With this model, the epistatic interaction can be inferred by hypothesis testing whether the regression coefficient *γ* is equal to zero, which can be conducted by a likelihood ratio test. Furthermore, by enumerating all possible combinations of loci pairs and performing statistical tests, we are able to detect all epistatic interactions. However, because of the large number of loci in a genome-wide association studies, such an exhaustive search can hardly be practical, and a variable selection strategy should then be applied to reduce the search space.

On the other hand, in a metagenome-wide association study with the case-control design, one typically sequences the microbial community of a number of patients and normal individuals, obtains gene scaffold by assembling the sequencing data, mapping sequence reads to the scaffolds to obtain abundance levels of the genes, and applies statistical approaches to test whether the abundance level of such a gene is significantly different between the case and the control. In such a study, a microbial gene is used as a marker, analogous to a locus in a traditional genome-wide association study. However, there also exists significant difference between metagenome-wide and genome-wide association studies. For example, the number of markers in a metagenome-wide study can be as large as 4 million, while that in a genome-wide study is typically less than 1 million. Another more important difference is the property of markers. In a genome-wide study, markers are factors, while in a metagenome-wide study, markers are continuous variables. Obviously, the former difference requires more efficient approaches in a metagenome-wide study, while the latter difference suggests either the customization of methods in genome-wide study to facilitate the manipulation of continuous variables or the development of novel statistical methods that take the continuous nature of microbial genes into consideration.

**Figure 1:**
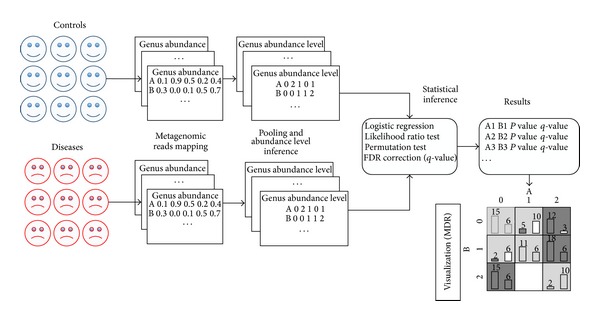
The overall procedure of metaBOOST: (1) obtain abundance through reads mapping; (2) infer abundance levels using Gaussian mixture model and EM algorithm; (3) identify possible epistatic interactions via logistic regression and permutation test; (4) validate identified epistatic interactions using MDR.

With the above analysis, we proposed in this paper the analysis of epistatic interactions between microbial genes. More specifically, we proposed a bioinformatics approach called metaBOOST that is designed based on a highly efficient epistasis detection algorithm named BOOST [19] and includes two extra steps: the discretization of abundance levels of microbial genes and a permutation test for accessing the statistical significance of candidate epistatic interactions. In detail, as shown in Figure 1, we first mapped metagenome sequencing reads to representative sequences of both microbial genus and KEGG orthologous (KO) groups to obtain abundance levels of genus and KO groups. Then, we fitted the distribution of the abundance levels to a mixed Gaussian distribution and estimate the associated parameters using an Expectation-Maximization algorithm. Next, we discretized continuous abundance to factors of at most three levels, analogous to genotypes in genome-wide association studies. Finally, we used a highly efficient algorithm in genome-wide study called BOOST to detect candidate interactions. In order to assess statistical significance of such candidate interactions, we further perform a permutation test and control the false discovery rate at a desired level.

### 2.2. Inference of Microbial Abundance Levels

The objective of this step is to obtain abundance levels of microbial genus or KEGG orthologous groups. In detail, given sequencing reads generated by a deep sequencing technology (typically illumina and 454), we mapped the reads to known microbial genes and summarized the number of mapped reads to obtain abundance levels of a microbial gene. The abundance levels are further normalized to illuminate the possible influence of different sequencing depths for different samples. Then, we made use of mapping between genes and genus to obtain genus abundance by summing up all of the abundance of genes corresponding to the same genus, in which gene length is considered and serves as divider since longer genes generate more reads. Similarly, we obtained KEGG orthologous (KO) abundance.

The abundance is continuous, ranging from 0 to 1 and in nature different from GWAS data. In order to utilize the methods developed for GWAS, we discretized the abundance data first into two or three levels, that is, {0,1} or {0,1, 2}. Particularly, in the two-level case, 0 represents nonexistence and 1 existence. In the three-level case, 0 represents nonexistence, 1 low-level existence, and 2 high-level existence. In our method, the discretization is performed automatically by fitting abundance levels of both case and control populations to a Gaussian mixture model and estimates the parameters using an Expectation-Maximization algorithm. And there exists two strategies for determining the number of levels for abundance data. First, we can use some objective criteria such as Bayesian information criterion (BIC). Second, we can use some empirical and heuristic strategies such as discarding a level that contains mainly zeros. In our paper, we adopt the latter for the purpose of seeking for simplicity.

### 2.3. Detection of Microbial Epistatic Interactions

The most popular method to detect epistasis is likelihood ratio test or logistic regression, in which two models are considered, that is, model with interaction term and the model without interaction term, such as the following:

 without interaction,
  (2) $$\begin{array}{c} \log\frac{P\left(Y=1\right)}{P\left(Y=0\right)}=\mu +\alpha X_{1}+\beta X_{2}, \end{array}$$
 with interaction:
  (3) $$\begin{array}{c} \log\frac{P\left(Y=1\right)}{P\left(Y=0\right)}=\mu +\alpha X_{1}+\beta X_{2}+\gamma X_{1}X_{2}. \end{array}$$

After fitting the above two models, we calculated the likelihood ratio or log-likelihood difference and this score is object to chi-squared distribution. A *P* value can then be computed and serves as a measurement of the strength of the epistatic interaction. Considering that the theoretical *P* value derived from chi-squared test may not reflect the true null distribution, we further use permutation test as described briefly in Procedure 1 to obtain simulated *P* value and derive *q*-value to control false discovery rate. Here, we adopted BOOST for the calculation of *P* value since this method was validated for its power and speed recently [24].

### 2.4. Visualization and Validation

After the above two steps, we obtained a list containing potential epistasis with correspond *q*-values. We then adopted the strategy used in multifactor dimension reduction (MDR) [15] to supply figures for visualizing patterns of epistasis detected. Briefly, MDR include four steps. (1) Select possible associated factors or factors under investigation. (2) List all possible combinations of the factors selected before in two or higher dimensions and evaluate the relative ratio in a model. (3) In order to assess every model in more accurate manner, cross-validation (usually tenfold) is used and average classification error is computed. (4) A list of all possible interaction models with their errors is obtained.

In general, there exist two strategies for validation. First, we can validate detected epistatic interactions by using independent computational methods. Second, we can perform validation through an experimental way, such as using extra samples to check the statistical significance of our findings. As for the former, systematic comparisons between different computational methods have been carried out intensively in literature [24, 25], and thus we think it is unnecessary to repeat such work in our paper. As for the latter, considering that an experiment is costly, time-consuming, and beyond the theoretical purpose of our paper, we would like to leave this possibility to some future projects.

## 3. Results

### 3.1. Data Sources

In simulation studies, we relied on existing benchmark datasets for epistatic detection to generate artificial data for evaluating the effectiveness of our method. Briefly, Velez et al. explored 70 models with different penetrance functions and generated a total of 42,000 simulated datasets [26]. From this resource, we selected a small number of 10 models and generated two datasets of 200 and 400 samples for each model. In each dataset, a total of 1000 loci (998 random and 2 of epistatic interaction) were generated. To simulate abundance levels, we further resorted to a Gaussian mixture model as detailed in the next section. Finally, we obtain 2 datasets (200 and 400 samples) for each of the 10 epistatic models.

For real metagenome-wide association studies, we selected two real datasets published recently [2, 27]. In detail, as part of the MetaHIT project, Qin et al. sequenced faecal samples of 124 European individuals and studied the impact of gut microbes on human health [27]. Among those individuals, 99 were infected with inflammatory bowel disease and the others were not. After metagenomic sequencing on the illumina platform, 576.7 Gb of sequence reads were generated, and each individual owns 4.5 Gb of sequence reads on average. Then, those huge amounts of reads were assembled into contigs by a de Bruijn graph-based method called SOAPdenovo [28]. Then, the metagene was used to predict long ORFs (longer than 100 bp) and those nonredundant ORFs (3.3 million in total) were considered as “genes” of microbes [29]. Next, those ORFs were mapping into reference microbial genomes to obtain microbial genus abundance and KEGG orthologue groups abundance. Another real metagenome-wide association studies dataset was generated by similar procedures [2]. In this study, 368 Chinese individuals' stool samples were sequenced and 1209.2 Gb of sequence reads was generated. Those individuals contain 183 patients of type 2 diabetes and 185 normal controls. After assembling and metagenomic gene prediction, 4,267,985 predicted genes were obtained and after mapping, 6,313 KEGG orthologues were obtained.

### 3.2. Simulation Studies

Existing metagenomics simulator such as MetaSim [30] can simulate sequencing data for metagenomics but cannot embed possible epistatic interactions in the simulated data. Existing methods for simulating genome-wide association studies can simulate case-control data but cannot embed epistatic interaction patterns into metagenomics data. Therefore, we proposed a simple method based on Gaussian mixture model to simulate abundance levels of microbial markers such as genus and KO. In detail, we adopt a two-step procedure as described below.

We generated epistatic data in continuous case based on discrete case, as formulated below:

(4) yi={0xi=0sampling  from  Normal(μ1,σ1)  xi=1sampling  from  Normal(μ2,σ2)xi=2,

where *x*_*i*_ denotes genotype data generated by simulator and *y*_*i*_ corresponding abundance levels.

**Figure 2:**
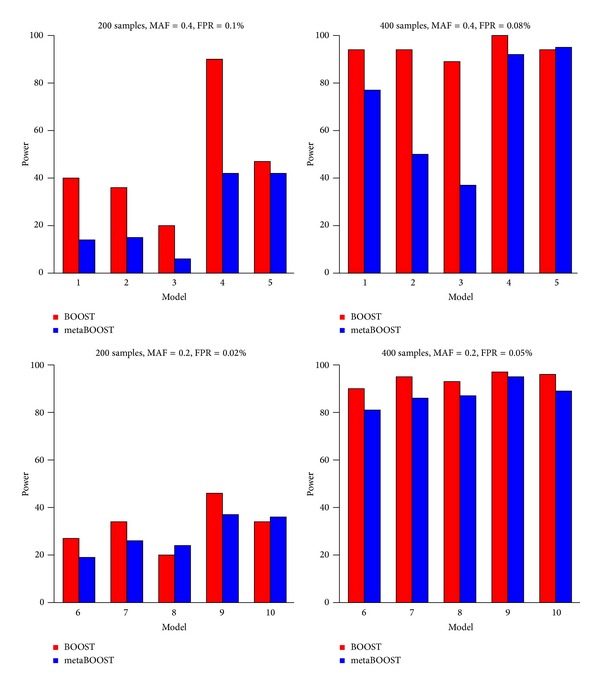
The results of simulation studies. We compare BOOST and metaBOOST on 10 epistatic interaction models. 100 datasets, each of 1000 markers, are generated for each model. The power is defined as proportion of identify the true epistatic interaction successfully.

After the generation of simulated data, we applied BOOST to identify embedded epistatic interactions in the original case-control data and metaBOOST to identify epistatic interactions in the simulated metagenomic case-control data, and we present results in Figure 2, in which the power of a method on a model is defined as the proportion of the ground-truth epistatic interactions being identified. From the figure, we first see clearly the effectiveness of metaBOOST in detecting epistatic interactions embedded metagenomic case-control data. For example, with 400 samples and an MAF of 0.2 and an FPR of 0.06% (subplot D), the power of metaBOOST for the five interaction models ranges from 0.80 to 0.90, suggesting the successful identification of the embedded interactions. Second, we observe that the power of metaBOOST for metagenomic data is in general not as high as BOOST for genomic data. This phenomenon is easy to understand because the extra step of converting continuous abundance levels to discrete factors by fitting the Gaussian mixture model may introduce extra noises in the data, thus resulting in the loss of the detecting power. Finally, we observe that the power of metaBOOST depends heavily on the number of samples. More specifically, with 200 samples, epistatic interactions embedded in model 3 can hardly be detected (power < 10% for MAF = 0.4 and FPR = 0.1%), while with 400 samples, epistatic interactions embedded in model 3 can reach about 40% (for MAF = 0.4 and FPR = 0.1%).

### 3.3. Genus Epistasis for Type II Diabetes

With the power of metaBOOST being verified by the simulation studies, we applied this method to a real metagenome-wide case-control dataset of gut microbe of 368 Chinese individuals [2], including 183 patients of type II diabetes and 185 healthy individuals.

**Figure 3:**
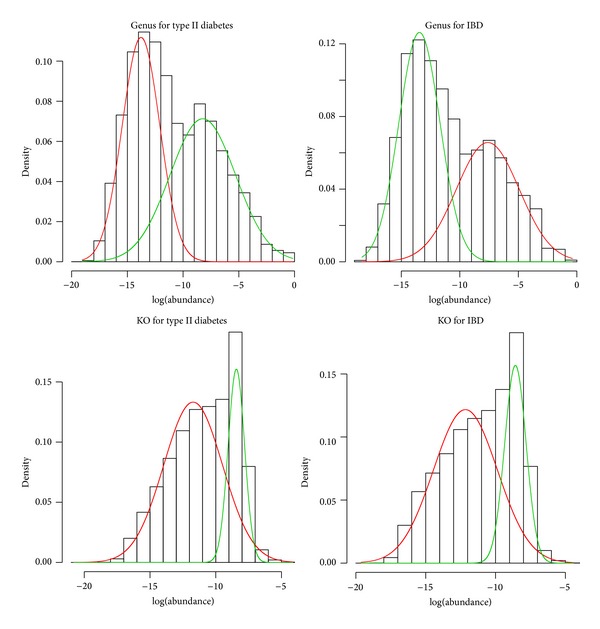
The distribution of abundance levels of genus and KOs for type 2 diabetes dataset and IBD dataset. The red and green curve are modeled by Gaussian mixture model and computed by EM algorithm.

We first fitted the distribution of abundance levels of genus of both the case and the control populations using a Gaussian mixture model and find that a two-component model fits the data very well (Figure 3) except for nonexistence. We then discretized abundance levels of genus to three categories. Because the number of genuses is not large, we enumerated all pairwise interactions of the genus and fitted a logistic regression model for each pair. We further performed the permutation test 1,000,000 times to estimate a *P* value for each pair and derive a *q*-value to characterize the statistical significance. Finally, we list top 10 interactions of the smallest *q*-values in Table 1.

In the list, the interaction between* Bifidobacterium* and* Actinobacillus* is reported with a very significant *q*-value (<10^6^). By reviewing the literature, we find that this potential epistatic interaction has been supported by several existing studies. For example, in literature [10, 31, 32],* Bifidobacterium* was reported to be associated with type 2 diabetes. The increase in abundance level of this genus will improve high-fat-diet-induced diabetes in mice, and such phenomenon shows significant difference between type 2 diabetes and healthy people. Besides, in literature [33],* Actinobacillus* was suggested to be associated with type II diabetes. Therefore, it is reasonable to assume that the interaction between* Actinobacillus* and* Bifidobacterium* may further increase the risk of carrying type 2 diabetes.

**Figure 4:**
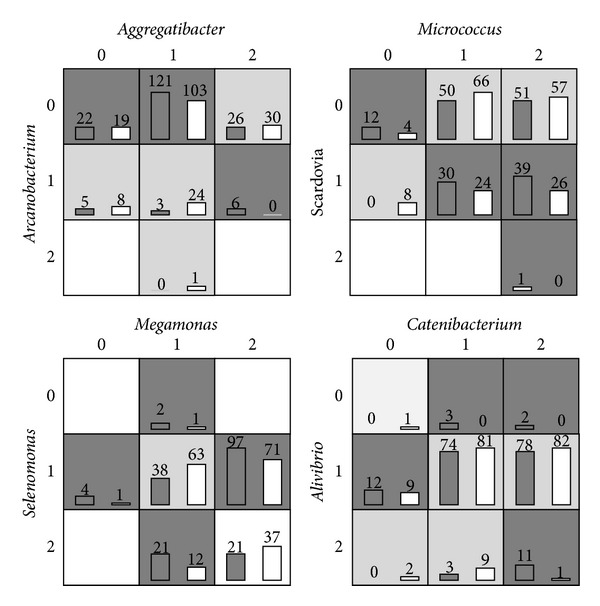
Four potential epistatic interactions between genuses in the type II diabetes dataset.

We then plot four possible interaction patterns generated by MDR in Figure 4. From the figure, we can clearly see the interaction patterns between two genuses. For example, in the left bottom subfigure, the disease risk is low if both of the abundance levels of* Megamonas* and* Selenomonas* are low or high and otherwise the disease risk is high. Similar interactions pattern can also be found in other subfigures.

### 3.4. Genus Epistasis for IBD

We also applied metaBOOST to another real metagenome-wide case-control dataset of gut microbe of 124 European individuals [27], including 25 patients of inflammatory bowel disease and 99 individuals without this disease. We first resorted to the same procedure as the above section to obtain abundance of microbial genus. Then, we used Gaussian mixture model to fit the distribution of abundance of genus of both the case and the control and Figure 3 shows that two-component model is also a good choice for this dataset. So, we discretized the abundance into low and high levels corresponding to the two components in the GMM plus nonexistence, which leads to three categories. The same logistic regression model and permutation test are used to output *P* value and *q*-value for each pair of genuses, and we list top 10 interactions with the smallest *q*-values in Table 2.

In the list, the interaction between* Lautropia* and* Thauera* is reported to have the smallest *P* value (4*e* − 6) and the smallest *q*-value (0.127). By reviewing literature, we find that both of* Lautropia* and* Thauera* belong to Proteobacteria, which is reported to be associated with IBD in [34] and the dysbiosis of Proteobacteria can result in IBD. So, it is reasonable to think that the interaction between* Lautropia* and* Thauera* may play an important role in the process of dysbiosis and IBD.

**Figure 5:**
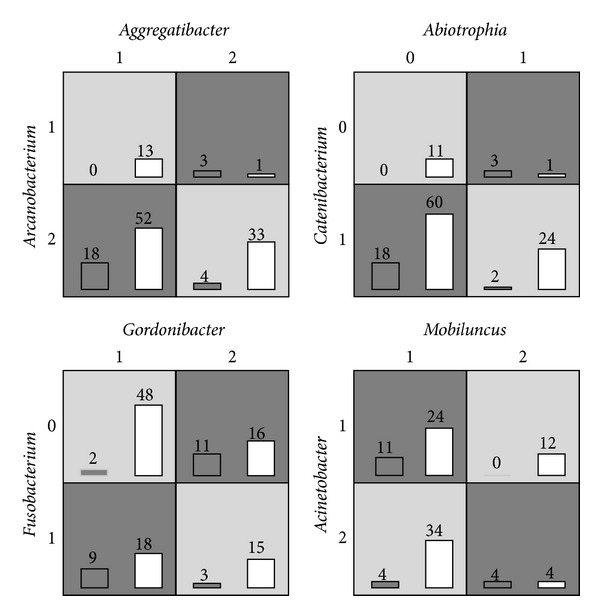
Four potential epistatic interactions between genuses in the IBD dataset.

We then plot four potential interaction patterns generated by MDR in Figure 5. Different from above section, we do not plot all three levels of each genus but only the two of three with significant number of samples and the remaining cells were discarded because of too little samples. But this does not affect the patterns themselves and we can still discover important interaction patterns from here. For example, in the left top subfigure, the disease risk is low if both of the abundance levels of* Slackia* and* Cellulosilyticum* are high or low, and the disease risk increases significantly otherwise. And as another example at the right bottom subfigure, the disease risk is high when both of the abundance levels of* Mobiluncus* and* Acinetobacter* are low or high, and the disease risk decreases otherwise.

### 3.5. KEGG Orthologous Epistasis for Type II Diabetes

Besides genus, functional annotation such as KEGG orthologues groups is also important for understanding the functions of human gut microbiome [2]. After having gene reference or catalogue of the 368 samples, functional annotation using KEGG and eggNOG database was performed. Finally, 6,313 KEGG orthologues (KO) are identified, which covered 47.1% and 60.9% of gene catalogue, respectively. And corresponding read counts can be converted into the abundance levels of microbial KOs.

**Figure 6:**
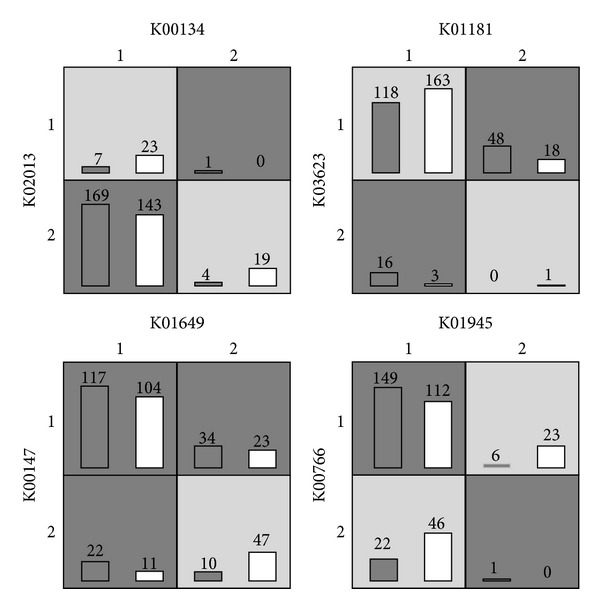
Four potential epistatic interactions between KOs in the type II diabetes.

We then applied metaBOOST only on KEGG orthologue groups or KO since more annotation information can be retrieved. Similarly, we fitted the abundance of KO using Gaussian mixture model with EM algorithm, and Figure 6 tells us that the two-component model is a relatively good choice. Here, we have to adopt a stepwise strategy since the number of KOs is 6,313 and the number of combinations of KO pair would be as high as ~2 million. First, we fit logistic regression models with only one KO and compute its *P* value. The *P* value can measure the effect of one KO on disease and we only select those 265 KOs with *P* value < 0.1. Then we enumerate all pairwise interactions of the selected KO and fit a logistic regression model for each pair. We further perform the permutation test 100,000 times to estimate a *P* value for each pair and derive a *q*-value to characterize the statistical significance. Finally, we list top 10 interactions of the smallest *q*-values in Table 3.

In the list, all interactions are reported to have small *P* values (≤2*e* − 5) and small *q*-values (≤0.06). But to the best of our knowledge, we find no literature to associate KOs with type 2 diabetes. Then, we map KOs into pathways using KEGG database and find 5 of 10 epistatic interactions that can share the same pathways as Table 4. If two KOs or two genes are involved in the same pathway, it is reasonable to think they may interact with each other in some way. And those common pathways are ko01230 (biosynthesis of amino acids), ko01110 (biosynthesis of secondary metabolites), and ko01100 (metabolic pathway). Obviously, those pathways are associated with biosynthesis and metabolic activities, which are important for microbes' life.

We then plot four potential interaction patterns generated by MDR in Figure 6. Again, we only plot two of three abundance levels with enough samples and omit those cells with small samples. From the figure, we can discover some obvious interaction patterns. For example, in the left bottom subfigure, the disease risk is low only when both of the abundance levels of K01649 and K00147 are high. This may tell that K01649 and K00147 can cooperate with each other to prevent type 2 diabetes. As another example, in the tight top subfigure, omitting the cells containing only 1 sample, we find that the disease risk is low only when both of the abundance levels of K01181 and K03623 are low. This may tell that K01181 and K03623 are both harmful for type 2 diabetes and increase in one of them can result in high disease risk.

### 3.6. KEGG Orthologous Epistasis for IBD

We then applied the same procedure as detailed in the above sections to analyze the IBD data. We mapped genes in the gene catalogue of the 124 European samples into the KEGG database to obtain the abundance of KEGG orthologue groups. We also fitted the distribution of abundance using Gaussian mixture model and we can see that the two-component model is also a good choice from Figure 6. So we discretized abundance levels of KO to three categories. Here, we also adopted a stepwise strategy to select significant KOs using one-variable logistic regression firstly, followed by epistatic interactions detection with two-variable logistic regression. Permutation tests (100,000 times) are also used to estimate a *P* value for each pair and derive a *q*-value to characterize the statistical significance. Finally, we list top 10 interactions of the smallest *q*-values in Table 5.

**Figure 7:**
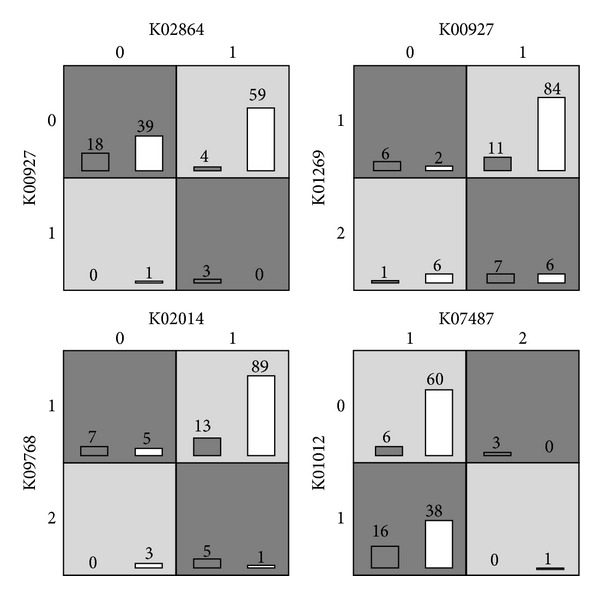
Four potential epistatic interactions of KOs in the IBD dataset.

In the list, only the first two pairs have significant small *q*-value (<0.05). After mapping into pathways, we cannot find common pathways in those KOs. We then plot four potential interaction patterns generated by MDR in Figure 7. From the figure, we can see that at least two of four cells have small samples, such as only 1 and 3 samples in the left top subfigure. This tells that the epistatic interactions between KOs are weak or not significant. The possible reasons may be the following: (1) the number of samples is small and the number of IBD patients is just 25 and (2) the epistatic interactions of KOs in this IBD dataset are hard to be identified.

## 4. Conclusions and Discussion

In this paper, we proposed a method called metaBOOST to detect epistatic interactions in metagenome-wide association studies. We first resorted to a Gaussian mixture model to automatically discretize abundance levels of microbial genus and microbial genes to categorical values and then relied on a logistic regression model to detect epistatic interactions at the genus and KO level. Results not only show the effectiveness of our approach in simulation studies but also suggest the existence of several potential epistatic interactions between microbial biomarkers in two real datasets of human gut microbial communities. The merit feature of our approach is the automatic discretization of abundance levels of microbial genus and genes. As one of the main differences between metagenome-wide and genome-wide association studies, the continuous form of microbial biomarkers brings the main difficulty for detecting epistatic interactions between such markers and makes the discretization step the prerequisite. Resorting to the Gaussian mixture model, as we have done in this paper, is certainly an effective way.

Certainly, our method can further be extended from the following aspects. First, since the understanding of epistatic interaction is not unique, the exploration of epistatic interactions between microbial biomarkers under different definitions will be necessary. Second, although our method of discretizing continuous abundance levels of microbial biomarkers has been demonstrated to be effective, it is still worth pursuing to directly build a statistical or machine learning method that is capable of detecting epistatic interactions between numeric-valued markers. The main difficulty is that the huge number of markers in a metagenome-wide association study prevents the exhaustive search of combinations of microbial markers, and the continuous form of such markers prevents the application of highly efficient computational tricks such as bit-wise operations that have been adopted in existing methods in genome-wide studies.

## Figures and Tables

**Procedure 1:**
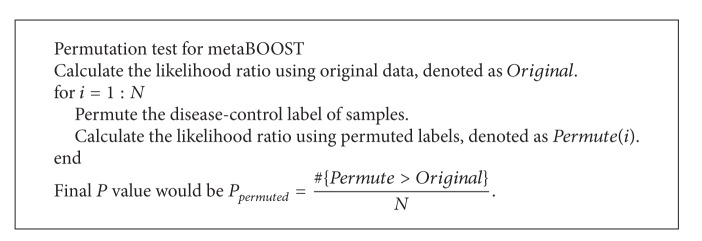
Procedure for the permutation test.

**Table 1:** Top 10 candidate genus epistatic interactions in the type II diabetes dataset.

Genus A	Genus B	*P* value	*q*-value
*Bifidobacterium *	*Actinobacillus *	0	0
*Aggregatibacter *	*Arcanobacterium *	6*e* − 6	0.084
*Pyramidobacter *	*Proteus *	8*e* − 6	0.084
*Acidaminococcus *	*Ureaplasma *	2*e* − 5	0.119
*Rhizobium *	*Veillonella *	2.3*e* − 5	0.119
*Micrococcus *	*Scardovia *	2.4*e* − 5	0.119
*Megamonas *	*Selenomonas *	2.8*e* − 5	0.119
Burkholderiales	*Abiotrophia *	3*e* − 5	0.119
*Kingella *	*Desulfotomaculum *	3.5*e* − 5	0.123
*Catenibacterium *	*Aliivibrio *	4.2*e* − 5	0.133

**Table 2:** Top 10 candidate genus epistatic interactions in the IBD dataset.

Genus A	Genus B	*P* value	*q*-value
*Lautropia *	*Thauera *	4*e* − 6	0.127
*Slackia *	*Cellulosilyticum *	1.3*e* − 5	0.206
*Abiotrophia *	*Catenibacterium *	3.6*e* − 5	0.292
*Streptobacillus *	*Anaerofustis *	4*e* − 5	0.292
*Edwardsiella *	*Peptoniphilus *	6.3*e* − 5	0.292
*Gordonibacter *	*Fusobacterium *	6.7*e* − 5	0.292
*Mobiluncus *	*Acinetobacter *	8*e* − 5	0.292
*Enterobacter *	*Thermoanaerobacter *	8.3*e* − 5	0.292
*Lautropia *	*Ureaplasma *	8.3*e* − 5	0.292
*Corynebacterium *	*Leptotrichia *	1.03*e* − 4	0.305

**Table 3:** Top 10 candidate KO epistatic interactions in the Type II diabetes dataset.

KO A	KO B	*P* value	*q*-value
K01760	K00948	0	0
K00134	K02013	0	0
K00134	K01756	0	0
K01181	K03623	0	0
K07487	K00811	0	0
K01649	K00147	0	0
K01945	K00766	1*e* − 5	0.04
K03427	K11928	1*e* − 5	0.04
K01945	K00968	2*e* − 5	0.06
K05808	K01808	2*e* − 5	0.06

**Table 4:** Common pathways involved in KO epistatic interactions in the type II diabetes dataset.

KO A	KO B	Common pathways
K01760	K00948	ko01230, ko01110, and ko01100
K00134	K01756	ko01110, ko01100
K01649	K00147	ko01230, ko01100
K01945	K00766	ko01110, ko01100
K01945	K00968	ko01100

**Table 5:** Top 10 candidate KO epistatic interactions in the IBD dataset.

KO A	KO B	*P* value	*q*-value
K02864	K00927	0	0
K07023	K06881	0	0
K02967	K11921	1*e* − 5	0.136
K00927	K03760	2*e* − 5	0.204
K09768	K10041	4*e* − 5	0.326
K00927	K01269	8*e* − 5	0.466
K07487	K03186	8*e* − 5	0.466
K02014	K09768	1.1*e* − 4	0.560
K07487	K01012	1.4*e* − 4	0.593
K03390	K00793	1.6*e* − 4	0.593
